# Outcomes for patients implanted with a cardioverter-defibrillator at <19 years of age: a Swedish national study

**DOI:** 10.1093/europace/euaf317

**Published:** 2025-12-11

**Authors:** Camilla Wirestrand, Fredrik Gadler, Annika Rydberg, Anders Nygren, André Rudolph, Anna Wålinder Österberg, Eva Fernlund, Ida Jeremiasen, Ingegerd Östman-Smith, Jenny Alenius Dahlqvist, Håkan Eliasson

**Affiliations:** Department of Women’s and Children’s Health, Karolinska Institutet, Tomtebodavãgen 18A, 17177 Solna, Sweden; Department of Medicine, Karolinska Institutet, Stockholm, Sweden; Department of Cardiology, Karolinska University Hospital, Stockholm, Sweden; Department of Clinical Sciences, Pediatrics, Umea University, Umea, Sweden; Department of Pediatric Cardiology, Sahlgrenska University Hospital, Gothenburg, Sweden; Department of Women’s and Children’s Health, Karolinska Institutet, Tomtebodavãgen 18A, 17177 Solna, Sweden; Department of Pediatric Cardiology Stockholm-Uppsala, Karolinska University Hospital, Eugeniavãgen 23, Solna SE-171 64, Sweden; Crown Princess Victoria Children’s Hospital, Linköping University Hospital, Linköping, Sweden; Division of Pediatrics, Department of Biomedical and Clinical Sciences, Medical Faculty, Linköping University, Linköping, Sweden; Crown Princess Victoria Children’s Hospital, Linköping University Hospital, Linköping, Sweden; Division of Pediatrics, Department of Biomedical and Clinical Sciences, Medical Faculty, Linköping University, Linköping, Sweden; Department of Clinical Sciences, Lund University, Lund, Sweden; Department of Clinical Sciences, Lund University, Lund, Sweden; Pediatric Heart Center, Skane University Hospital, Lund, Sweden; Department of Pediatric Cardiology, Sahlgrenska University Hospital, Gothenburg, Sweden; Institute of Clinical Sciences, Sahlgrenska Academy, Gothenburg University, Gothenburg, Sweden; Department of Clinical Sciences, Pediatrics, Umea University, Umea, Sweden; Department of Women’s and Children’s Health, Karolinska Institutet, Tomtebodavãgen 18A, 17177 Solna, Sweden; Department of Pediatric Cardiology Stockholm-Uppsala, Karolinska University Hospital, Eugeniavãgen 23, Solna SE-171 64, Sweden

**Keywords:** Implantable cardioverter-defibrillator, Children, Cardiac arrhythmias, Sudden cardiac death

## Abstract

**Aims:**

To explore the prevalence and incidence of paediatric implantable cardioverter-defibrillator (ICD) patients in Sweden and identify risk factors associated with appropriate shocks and adverse events.

**Methods and results:**

We performed a nationwide, retrospective cohort study of ICD use in paediatric patients (<19 years) between 1995 and 2017; 120 patients underwent ICD implantation at median age 14.7 (range 1.1–18.9) years and were followed for 7.1 (0.3–20.4) years. Fifty-four patients (45%) received a primary preventive ICD; 46% had cardiomyopathy, and 41% had primary electrical disease. The estimated 5-year survival without appropriate shocks was 68% (confidence interval 59–78). Secondary preventive ICDs and lower weight (<30 kg) at implantation were associated with a higher rate of appropriate shocks, respectively. Lower weight at implantation was not a risk factor for adverse events. Inadequate medication and insufficient compliance were common in patients who experienced shocks. Less than half (46%) of the cases with inappropriate shocks were adequately medicated with good compliance. Secondly, an incidence and prevalence study of all paediatric and adult patients who had an ICD implanted in Sweden was analysed in 4-year periods between 2002 and 2021. The incidence of paediatric ICD implantations in Sweden peaked at 0.56 per 100 000 person-years in 2010–13, decreasing to 0.45 per 100 000 person-years in the last study period (2018–21).

**Conclusion:**

Appropriate shocks were more than twice as common in the youngest patient group, whereas adverse events were not more frequent than in the older patient group. Inadequate medication and lack of compliance were common in connection with ICD shocks.

## Graphical Abstract

**Graphical Abstract:**
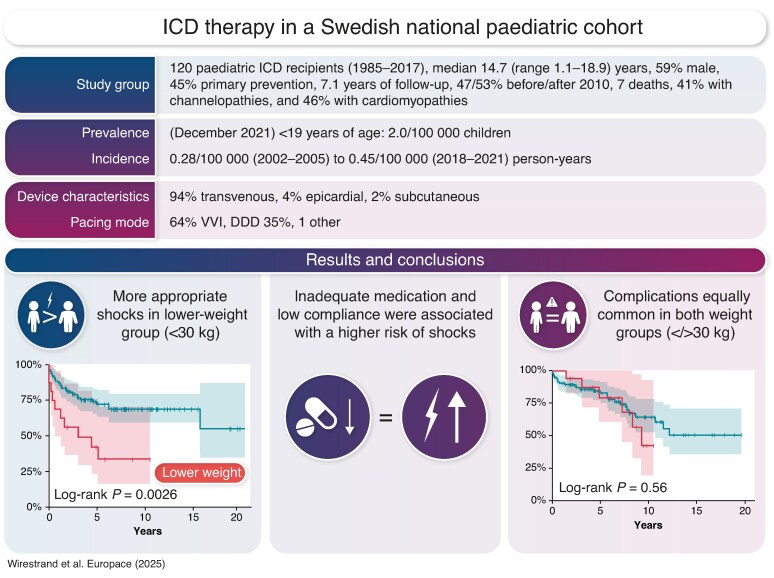

What’s new?

- We present unique incidence and prevalence data of young implantable cardioverter-defibrillator carriers with nationwide coverage.
- The youngest patients (lower-weight group) did not have a higher risk of inappropriate shocks or complications.
- Poor drug compliance and inadequate doses of antiarrhythmic medication were more common than expected.

## Introduction

Implantable cardioverter-defibrillators (ICDs) are essential tools for treating malignant arrhythmias and preventing sudden cardiac death (SCD) in high-risk populations.^1^ According to current guidelines, there is consensus, irrespective of age, to implant an ICD in survivors of aborted cardiac arrest when it can be assumed that the underlying cause is not reversible.^2–5^ Who should be a candidate for a primary prevention ICD implantation is less clear, especially in the paediatric population. Recommendations largely depend on the underlying disease and associated risk factors.^6^ Risk stratification strategies are developing but are disease-dependent and challenging.^7^ Risk calculators may provide prognostic help to predict patients at risk, but the predictive value in paediatric patients appears to be low.^5,8^ It is well known that ICD implantation rates vary across Europe^9^ although no significant differences regarding cardiovascular morbidity have been observed.^10^ However, epidemiological data with prevalence and incidence calculations regarding paediatric ICD implantation rates in national cohorts are lacking.

Implantable cardioverter-defibrillator therapy is potentially life-saving but also carries a risk of adverse events, such as inappropriate shocks and complications, especially in the young.^11–15^ However, the results are conflicting, possibly due to a general underrepresentation of children in many studies, making any comparison between age groups challenging. Children are underrepresented in ICD studies mainly because malignant arrhythmias are more uncommon in this age group but possibly also on the presumption that the risk–benefit ratio is disadvantageous in younger children due to complications. Age-specific ICD complications have rarely been studied, but two studies, by Silvetti *et al*.^16^ and Dechert *et al*.,^17^ showed that complications were not more common in the paediatric patients. On the other hand, lead-related complications are reported to be more common in lower ages of pacemaker-treated children, so this remains an issue.^18,19^ However, even taking this into account, the scientific evidence for applying different criteria when considering an ICD implantation in a child is lacking, in our opinion.

Adequate antiarrhythmic medication and optimized compliance are essential to avoid the risk of both appropriate and inappropriate ICD shocks. However, the prevalence of good compliance and adequate antiarrhythmic medication in connection with ICD therapy in the young has only rarely been studied.^20,21^ To address this knowledge gap, we performed a nationwide, retrospective cohort study of young ICD-treated patients to (i) identify risk factors associated with adverse events or appropriate shocks, (ii) study the prevalence and incidence of young ICD carriers in Sweden, and (iii) investigate compliance with antiarrhythmic medication in correlation to appropriate and inappropriate events. We hypothesized that low weight and younger age at ICD implantation were risk factors associated with adverse events and appropriate shocks.

## Materials and methods

### Study design and patient selection

This is a retrospective, multicentre, nationwide study. All patients aged <19 years who underwent ICD placement at any hospital in Sweden between 1995 and 2017 were retrospectively identified by searching the Swedish ICD and Pacemaker Registry (https://www.pacemakerregistret.se), a national registry established in 1989, that comprises all ICD implantations in Sweden. The registry has nearly 100% coverage of ICD implantations. Any identified patient aged <19 years with an ICD, irrespective of underlying cardiac diagnosis, was considered eligible for the study. Identified patients were asked to participate, and they or their parents provided written consent. A total of 140 patients aged <19 years at the time of ICD implantation were identified. Eighteen patients did not respond to at least two letters, one patient declined participation, and one further patient was unreachable; these 20 patients (14% of the cohort), with a median age of 15 years at ICD implantation, were therefore excluded from further analyses. All the excluded patients were alive at the time of eligibility evaluation. The study was approved by the Ethics Committee at Norrland’s University Hospital.

Additionally, we performed (i) a prevalence study of ICD carriers in Sweden by searching the Swedish ICD and Pacemaker Registry, including all alive patients carrying an ICD by 31 December 2021, and (ii) an incidence study of all ICD implantations in Sweden, from 2002 to 2021. We compared prevalence and incidence rates in patients aged <19 years (young) vs. ≥19 years at implantation. This was a separate study where all identified patients were included.

### Data collection

The final study population for the analyses of risk factors consisted of 120 patients with an ICD implanted at <19 years of age for primary prevention (no documented life-threatening sustained ventricular arrhythmia (VA) or sustained VA without syncope) or secondary prevention (arrhythmogenic syncope due to VA or cardiac arrest). Follow-up (FU) data were collected up to 31 December 2018, including all patients who had entered the adult age range. Data were reviewed and extracted retrospectively from clinical charts and the Swedish ICD and Pacemaker Registry. Data regarding implant indication, age, sex, height, and weight at implantation, as well as medical history including clinical presentation, diagnoses, cardiac function, genetic testing results, and medication use, were collected. Prophylactic antiarrhythmic therapy, including beta-blockers, was evaluated with respect to dose (adequate) and compliance with medication, especially in the occurrence of an ICD shock. All data were extracted from patient records, and if the specific information was missing, the patient was excluded from further analyses regarding medication in connection with shocks. Compliance with medication was not presumed but had to be explicitly stated. An *inadequate dose* was defined as a dose falling below the lowest recommended dose range for any specific antiarrhythmic medication prescribed for the patient, regardless of indication.^22^ We did not evaluate whether the chosen antiarrhythmic treatment adhered to international guidelines.

The following ICD data were collected: age and date of ICD implantation, type (subcutaneous, epicardial, or endocardial), and mode (VVI, DDD, or CRT) of pacing, as well as other technical details, including data on re-interventions, and ICD system complications. Data on each ICD shock were collected and analysed. An *appropriate shock* was defined as a shock delivered for either ventricular tachycardia (VT) or ventricular fibrillation (VF); an *inappropriate shock* was defined as a shock delivered for any rhythm other than VT or VF. A *complication* was defined as any event leading to an unforeseen re-intervention of the ICD system. Battery depletion was not considered a complication. An *adverse event* was defined as either an inappropriate shock or a complication. For each event, the following data were analysed: ICD settings, medication (type, dose, and compliance), and type of arrhythmia. Implantable cardioverter-defibrillator rhythm strips were not always available in the patient charts; therefore, the definition of the occurring arrhythmia was based on provider documentation. For the analysis of *ICD system survival*, any event leading to re-intervention of the ICD system was considered an endpoint. We arbitrarily divided the cohort into an early cohort (ICD implantation before 2010; 57 patients) and a late cohort (ICD implantation after 2010; 63 patients) to detect differences over time. To analyse the impact of low age and small body size on outcome, body weight (<30 kg vs. ≥30 kg) was chosen as a proxy for age and body size. The Swedish Population Register, maintained by Statistics Sweden (www.scb.se), was used to analyse the incidence and prevalence of ICD carriers in Sweden in patients aged <19 years, relative to age-specific population data. The incidence and prevalence in this age group were compared with those of the adult population (aged ≥19 years) in Sweden for the same period.

### Statistics

Descriptive data and results are presented as counts, frequencies, proportions, median with range, or median with interquartile range (IQR) when specified. Multiple Cox hazard regression analyses were performed to evaluate the effects of selected variables on ICD system survival, time-to-appropriate shock, inappropriate shock, complication, and adverse event, respectively. Additionally, 5-year event-free survival estimates with confidence intervals (CIs) were calculated for variables mentioned above. An explantation of an ICD was not regarded as a re-intervention in the context of survival analyses, irrespective of the reason for the explantation (when the indication for ICD treatment no longer persisted, before heart transplantation, or after patient/parental decision). Independent predictors used were body weight (<30 kg vs. ≥30 kg, defined as lower-weight group vs. higher-weight group) at implantation, type of disease {primary electrical disease [PED] [long QT syndrome (LQTS), catecholaminergic polymorphic ventricular tachycardia (CPVT), Brugada syndrome, or idiopathic VT/fibrillation], cardiomyopathy, other}, type of indication (primary or secondary), and the time bracket (<2010 vs. ≥2010) at implantation. Kaplan–Meier curves were used to estimate survival times (time to event). Five-year survival tests were calculated with a 95% CI. A hazard ratio (HR) was calculated for each covariate. A *P*-value of <0.05 was considered significant. Fisher’s exact test was used to compare the prevalence of inappropriate shocks and adverse events in the lower-weight group vs. higher-weight group. For prevalence analyses, all alive patients in Sweden by 31 December 2021, with an ICD implanted, were included, and age-specific prevalence measures (per 100 000 alive persons) were calculated in the age groups <19 years (young) vs. ≥19 years (adults). For incidence calculations, the study period (1 January 2002 to 31 December 2021) was divided into five 4-year periods to capture changes over time. The average incidence rate (implants per 100 000 person-years) was calculated for each year and then averaged for every 4-year period. To compare the incidence rates in young and adult patients, an incidence rate ratio (IRR) was calculated by dividing the incidence (per 100 000 person-years) in patients aged ≥19 (=Iadult) by patients aged <19 years (=Iyoung) at ICD implantation: IRR = Iadult/Iyoung.

## Results

### Prevalence and incidence of implantable cardioverter-defibrillator therapy in Sweden

**Figure 1:**
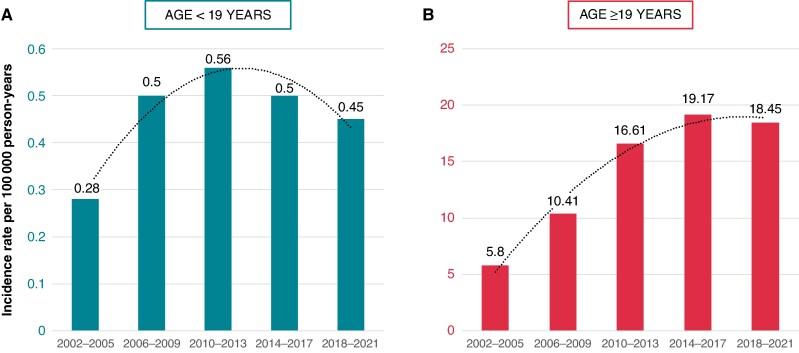
Age-specific incidence rate of new ICD implantations in the age groups <19 years (left panel *A*) and ≥19 years (right panel *B*) in Sweden. Patients are grouped by time periods, and each bar displays the average incidence rate for the years included in that period. Note the different scales of the *y*-axes between the age groups. ICD, implantable cardioverter-defibrillator.

*Figure 1A* shows that the yearly incidence rate of new ICD implantations in the age group <19 years in Sweden increased from 0.28 per 100 000 person-years in 2002–05 to 0.56 per 100 000 person-years in 2010–13, then decreased to 0.45 per 100 000 person-years in the last study period, 2018–21. In adults (≥19 years), the incidence rate increased over time to reach a plateau in the last study period (*Figure 1B*). The IRR in adults compared to those aged <19 years had doubled in the last study period (2018–21), compared to 2006–09 (see Supplementary material online, *Figure S1*). The prevalence of ICD carriers aged <19 years in Sweden by 31 December 2021 was 2.1 per 100 000 age-specific inhabitants, whereas the prevalence of ICD carriers aged ≥19 years was 172.2 per 100 000. The proportion of ICD implants in patients <19 years in 2018–21 was 0.69%.

### Baseline characteristics

**Figure 2:**
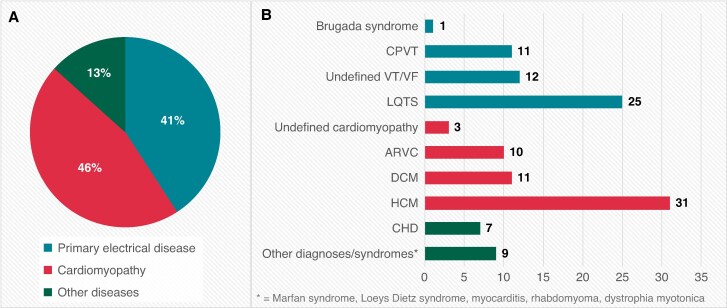
Patients grouped by disease categories (*A*) and specific diagnoses (*B*). ARVC, arrhythmogenic right ventricular cardiomyopathy; CHD, congenital heart disease; CPVT, catecholaminergic polymorphic ventricular tachycardia; DCM, dilated cardiomyopathy; HCM, hypertrophic cardiomyopathy; LQTS, long QT syndrome; VF, ventricular fibrillation; VT, ventricular tachycardia.

*Table 1* details the clinical characteristics. Between 1995 and 2017, an ICD was implanted in 120 patients (49 girls and 71 boys) aged <19 years with a median age of 14.7 (range 1.1–18.9) years. The median FU time was 7.1 (range 0.3–20.4) years, with no significant difference between weight groups. The median age at last FU was 21 (8–36) years. Sixteen (13%) of the patients had a body weight of <30 kg and an age of <12 years at the time of ICD implantation, and their characteristics are detailed in Supplementary material online, *Table S1*. Indications for ICD implantation were primary prevention in 54 patients (45%). Fifty-six (47%) patients received the ICD before 2010 (*Table 1*). *Figure 2* details the characteristics of the underlying disease. Forty-nine patients (41%) were diagnosed with PED, most commonly LQTS or CPVT. Fifty-five patients (46%) were diagnosed with cardiomyopathy.

**Table 1:** Characteristics of 120 patients at first ICD implantation

	Weight <30 kg *N* = 16	Weight ≥30 kg *N* = 104
Female/male	6 (38), 10 (62)	43 (41), 61 (59)
Time era		
<2010	7 (44)	49 (47)
>2010	9 (56)	55 (53)
Type of disease		
PED	7 (44)	42 (40)
Cardiomyopathy	9 (56)	46 (44)
Others	0 (0)	16 (15)
Indication for ICD therapy		
Primary	6 (38)	48 (46)
Secondary	10 (62)	56 (54)
ICD system		
Transvenous	12 (75)	101 (97)
Epicardial	4 (25)	1 (1)
Subcutaneous	0 (0)	2 (2)
Mode of pacing; transvenous and epicardial		
VVI	15 (94)	62 (60)
DDD	1 (6)	41 (39)
CRT	0 (0)	1 (1)
FU, duration	7.5 [4.8–10]	7 [3–10]

Values are number of cases (%) or years; median [IQR].

CRT, cardiac resynchronization therapy; FU, follow-up; ICD, implantable cardioverter-defibrillator; IQR, interquartile range; PED, primary electrical disease.

### Implant characteristics

Of the 120 patients, 113 (94%) received a transvenous system as their first ICD, five patients (4%) received an epicardial ICD, and two patients (2%) received a subcutaneous ICD (S-ICD) (*Table 1*). Six patients (5%) had switched to a different ICD system by the study’s endpoint compared to their first ICD; 115 patients had a transvenous system, three patients had a subcutaneous system, and two patients had an epicardial system. The first selected ICD pacing mode was VVI in 75 patients (64%) and DDD in 42 patients (36%), and one patient had CRT.

### Survival of the implantable cardioverter-defibrillator system and risk of re-interventions

One hundred eighteen re-interventions were performed in 64/120 patients (53%): a median of 1 (1–4) intervention per affected patient. Sixty patients underwent ICD system re-intervention, either as a planned re-intervention (such as battery depletion) or due to complications. In about half of the cases, battery depletion was the cause of the first intervention of the ICD system. Four patients had their ICDs explanted when the indication for ICD treatment no longer persisted, one of whom had prior interventions before the explantation. One patient had the ICD explanted due to a device complication and did not receive a new ICD after a parental decision to decline further treatment. Seven patients underwent heart transplantation and had their ICDs explanted. We observed a decreased survival time for the ICD systems in the early era (HR = 2.16; *P* = 0.012) compared to the late era. The disease category, ICD indication, or weight group did not significantly affect ICD system survival (see Supplementary material online, *Figure S3* and *Table S2*). The estimated 5-year intervention-free survival was 70% (CI 0.613–0.796).

### Appropriate shocks

**Figure 3:**
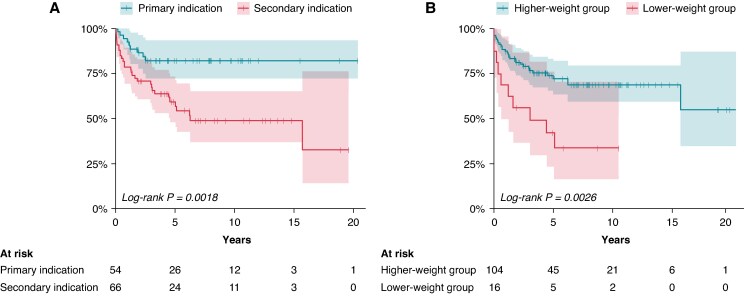
Probability of freedom from appropriate shocks. Patient groups are compared with respect to (*A*) indication for ICD therapy (primary vs. secondary) and (*B*) weight group (lower-weight group vs. higher-weight group). ICD, implantable cardioverter-defibrillator.

A total of 138 appropriate shocks were delivered in 39/120 patients (32%), with a median of 2 (1–18) shocks per affected patient. The first appropriate shock was delivered <1 year after the first ICD implantation in 18/39 patients (46%). The estimated 5-year survival without appropriate shocks in the total population was 68% (CI 0.59–0.78). Survival analyses (*Figure 3*) showed a higher shock rate in the lower-weight group (63% vs. 28%; HR 2.7; *P* = 0.008) and in the secondary prevention group (45% vs. 17%; HR 3.7; *P* = 0.001). Ten of 16 patients (62%) in the lower-weight group received their ICD as a secondary preventive measure, and 9/10 experienced appropriate shocks. Of the six patients with a primary preventive ICD in the lower-weight group, one patient (17%) experienced an appropriate shock. The type of disease or era (early/late) did not significantly affect the results (see Supplementary material online, *Table S3*).

### Inappropriate shocks, complications, and overall adverse events

Thirty-one inappropriate shocks occurred in 25/120 patients (21%), with a median of 1 (1–3) shock per affected patient. The first inappropriate shock was delivered <1 year after the first ICD implantation in 11/25 patients (44%). The estimated 5-year survival without inappropriate shocks was 84% (CI 77–91). Survival analyses showed that neither ICD indication, disease category, nor era significantly impacted the risk of inappropriate shocks (see Supplementary material online, *Figure S4* and *Table S4*). Survival analysis could not be performed for weight because the group did not meet the assumptions for proportional hazards, which is why Fisher’s exact test was performed instead. Two of 16 patients (12%) in the lower-weight group and 23/104 (22%) in the higher-weight group experienced one or more inappropriate shocks; this difference was not statistically significant. Commonly reported causes of inappropriate shocks were T-wave oversensing (26%), poor medication compliance (23%), non-malignant arrhythmias (23%), sinus tachycardia (13%), and lead dysfunction (6%).

**Figure 4:**
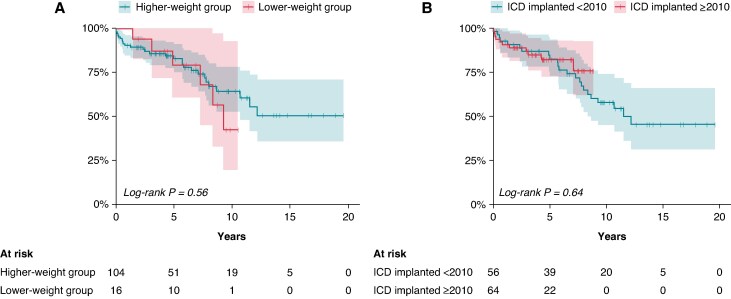
Probability of freedom from ICD-related complications leading to a surgical intervention. Patients are compared with respect to (*A*) weight group (lower-weight group vs. higher-weight group) and (*B*) cohort era (ICD implantation <2010 vs. >2010). ICD, implantable cardioverter-defibrillator.

There were 43 complications leading to re-interventions of the ICD system in 34/120 patients (28%): 1 (1–4) complication per affected patient, without significant differences between subgroups (*Figure 4* and Supplementary material online, *Table S5*). The estimated 5-year survival without complications was 82% (CI 0.75–0.90).

Lead dysfunction was the most common complication, 26/43 (60%), followed by infections, lead dislodgement, and device malfunction. Eight of nine (90%) of the complications in the lower-weight group, compared to 18/34 (53%) in the higher-weight group, were lead-related. Seventy-six adverse events (complications or inappropriate shocks) occurred in 48 (40%) of 120 patients; the median was 1 (1–4) adverse event per affected patient. The estimated 5-year survival without any adverse event was 71% (CI 0.63–0.80).

### Pharmacological treatment in connection with appropriate and inappropriate shocks

Adequate data regarding medication were present in 118/120 patients at the time of ICD implantation and in connection with 161/169 ICD events. Ninety-seven patients (81%) were prescribed antiarrhythmic medication at discharge after the ICD implantation. Adequate medication and good compliance were present in more than three-quarters (78%) of the overall occurring appropriate shocks (see Supplementary material online, *Figure S2A*). However, 16/38 of the patients with appropriate shocks had at least one shock with either inadequate/no medication or poor compliance. Similarly, regarding inappropriate shocks, adequate medication and good compliance were present in less than half of the shock episodes (see Supplementary material online, *Figure S2B*). Among the remainder, 5/28 shocks appeared without medication, 1/28 with inadequate medication dose, and 9/28 in non-compliance with medication. Thus, 15/28 inappropriate shocks appeared without adequate drug protection. Drug information was missing in three cases. Eleven of 23 patients had at least one inappropriate shock with either inadequate/no medication or poor compliance.

### Mortality

Seven patients with ICD included in the study died at a median age of 17 (11–36) years. In addition, one patient with CPVT, who had the ICD explanted at his request and, therefore, was no longer included in the study, died at age 21, presumably from a malignant arrhythmia (see Supplementary material online, *Table S6*). The seven patients still included in the study at their demise had cardiomyopathy or congenital heart disease. One patient with congenital heart disease died from multi-organ failure due to heart failure. Four patients with cardiomyopathy suffered a malignant arrhythmia that the ICD could not terminate. One further patient with cardiomyopathy died due to an infection that caused multi-organ failure, and one patient died suddenly abroad, where it was not possible to examine the ICD.

## Discussion

### Main findings

As expected, we observed a higher rate of appropriate shocks in the lower-weight group, possibly due to a higher clinical risk threshold for considering implantation in the youngest patients, because of the assumption of a higher risk of complications. However, unexpectedly, there was no higher rate of adverse events in the lower-weight group than in the higher-weight group. Furthermore, our study is the first to report prevalence and compliance with medication in connection with ICD shocks, and the results show a surprisingly high proportion of patients without adequate drug protection.

We also present unique incidence and prevalence data, showing that the incidence of ICD implants among individuals aged <19 years has decreased in Sweden over the last few years.

### Prevalence and incidence of implantable cardioverter-defibrillator implantations

Patient registries with a reasonably good coverage of ICD carriers are rare internationally. In Sweden, we have a national ICD registry with a near-100% coverage, enabling us to present reliable incidence and prevalence data of paediatric ICD carriers. We have not found published age-specific prevalence data of young, alive ICD carriers in other national cohorts for comparison. Interestingly, the proportion of paediatric ICD implantations has decreased over the last two decades. Between 2018 and 2021, paediatric implantations in Sweden accounted for 0.69% of the total number of ICD implantations. Data from the Danish pacemaker and ICD register (https://icddata.dk) showed that the proportion of young patients (<20 years) with an ICD implanted in 2022 was 0.9%.

### Survival of the implantable cardioverter-defibrillator system and risk of re-interventions

The survival of the ICD system reflects the total burden of interventions for ICD carriers. The disease category, ICD indication, or weight group did not significantly influence ICD system survival. We observed a shorter survival time in the early-era group (<2010) than in the late-era group, although the difference became apparent after ∼5 years, so the results should be interpreted cautiously. However, improved peri-procedural care, age, and/or type of ICD devices may have positively impacted the outcome in the late-era group, factors we did not examine in this study. The overall 5-year intervention-free survival in our study was 70%. In a study by Le Bos *et al*.,^23^ the 5-year intervention-free survival rates were 79 and 82% in patients with epicardial ICD vs. endocardial ICD, respectively. These numbers are similar to the 5-year intervention-free survival in our late-era group.

### Appropriate shocks

As hypothesized, we observed a higher rate of appropriate shocks in the lower-weight group (63%) compared to the higher-weight group (28%). This is possibly due to the individuals in the lower-weight group comprising a highly malignant phenotype, as low age at disease onset in many underlying conditions correlates with the occurrence of life-threatening arrhythmias. It is also possible that some individuals with a considerable risk for new arrhythmias, especially in primary prevention, were not considered candidates for an ICD implantation, on the assumption that the risk of adverse events is much higher in the youngest patients. In a recent study by the Danish group Thuraiaiyah *et al*.^13^ including children <15 years at the time of ICD implantation, 69% experienced appropriate shocks over a FU of 9 years.

Our estimated 5-year survival without appropriate shocks was 68%. Two studies reported similar 5-year survival estimates without appropriate shocks.^23,24^ The risk of appropriate shocks was significantly higher in the secondary prevention group than in the primary prevention group, at 45% vs. 17%, which aligns with previous studies.^14,25^  ^,26^ In contrast, the Danish ICD study by Thuraiaiyah *et al*.^13^ did not report a significant difference in appropriate shock rates between primary and secondary prevention ICDs. Differences between studies may, to some extent, be explained by inconsistencies in the definition of primary vs. secondary prevention. Our study found no difference in appropriate shocks when comparing disease categories.

### Inappropriate shocks, complications, and overall adverse events

The 5-year survival rate without inappropriate shocks in our cohort was 84%, and 21% of patients experienced at least one inappropriate shock. Von Bergen *et al*.^24^ reported a 5-year survival rate of 75% without inappropriate shocks. Einbinder *et al*.,^20^ with data collected from 2006 to 2020 and a FU of 5.6 years, reported a low prevalence of inappropriate shocks at 10%, mainly attributed to ICD programming. The prevalence of inappropriate shocks in our late-era group (>2010) was 11%, which may support the notion that recently improved programming algorithms decrease the risk of inappropriate shocks. However, a Danish study covering ICD implantations over a 40-year period also reported a low incidence of inappropriate shocks, corresponding to a 10-year cumulative incidence of 15%.^13^

The estimated 5-year survival without complications was 82% in our study, and 28% experienced at least one complication. Complication frequencies in previous studies with varying FU times ranged from 14 to 41%.^13,14,20,25,27,28^ Notably, we did not identify any differences in complication rates across subgroups, including the lower-weight group. Lead dysfunctions, a well-known cause of complications, especially in young ICD carriers,^29^ were the most common cause of complications in our study (60%), which compares well with the results of previous studies. It should be noted that 8/9 (90%) of the complications in the lower-weight group compared with 18/34 (53%) in the higher-weight group were lead-related, indicating that lead-related problems are more common in the lower age groups.

The estimated 5-year adverse event-free survival was 71% in our study, which compared well with that reported by DeWitt *et al*.,^11^ which also reported a 5-year adverse event-free survival of 71%. Adverse events are an interesting variable when comparing the potential life-saving effect of ICD therapy in relation to the patient’s quality of life when suffering inappropriate shocks and complications. Some studies report a higher prevalence of inappropriate shocks or complications in paediatric patients compared to adults or young adults.^11,12^ To our surprise, we did not observe a higher prevalence of adverse events in the lower-weight group than in the higher-weight group. We believe this finding is noteworthy, as it could contribute to a more equal and balanced evaluation of the decision to implant an ICD in a young individual. However, the lower-weight group in our study accounts for only a small part (13%) of the study population, so it is essential to interpret the results cautiously.

Over the last decade, S-ICD has increasingly become an alternative to epicardial or transvenous ICD therapy in patients without the need for anti-bradycardia or anti-tachycardia pacing, and the number of publications of paediatric cohorts is growing.^30^ The hope is to reduce lead-related complications without increasing the risk of inappropriate shocks. In a large adult study by Migliore *et al*.,^31^ the complication rate was low, but inappropriate shocks remain an issue. Published paediatric studies with relatively short FU intervals of 1.5–3.5 years still report adverse events in 21 to 30% of the cases.^21,30^ Our cohort included only 2% of S-ICD. The reason for this is likely that we have not included patients from 2018 onwards, but there is also possibly an influence from regional practices. Interestingly, in the recent Danish paediatric ICD cohort study, only one of 72 patients had an S-ICD.^13^

### Medication in connection with inappropriate and appropriate shocks

Poor compliance with medication is a well-known problem, especially in adolescents.^32^ It has, however, rarely been studied in young ICD patients, although compliance with (adequate) medication is a prerequisite to avoid both appropriate and inappropriate shocks. Less than half (46%) of registered inappropriate shocks were adequately medicated with good compliance, and 42% of the patients with appropriate shocks had at least one shock with either inadequate/no medication or poor compliance. Berul *et al*.^26^ acknowledged in their study that inappropriate shocks, not caused by lead problems, necessitated clinical interventions aimed at improving medication compliance or initiating antiarrhythmic therapy. Previous studies rarely accounted for compliance with the medical treatment in connection with ICD events. Our conclusion from the results of our study is that many ICD shocks, both appropriate and inappropriate, could be prevented with stricter review of patients’ treatment protocols and continuous work to improve compliance with prescribed medication. We recognize that our study does not allow explanatory analyses, but we believe our observations should encourage further prospective, large-scale studies.

### Strengths and limitations

The retrospective study design is a limitation. Differences in subgroups of patients may not have been detected due to the relatively small sample sizes, especially regarding the lower-weight group. Twenty (14%) of the 140 identified paediatric patients with ICD in Sweden did not actively consent to participate and were therefore excluded from the study on risk factors. However, all these patients were alive at the beginning of the data collection, and we have no reason to expect them to differ significantly from the included study population in terms of events or ICD survival. Furthermore, we included patients over a large time span, which may have affected the generalizability of the results. The youngest children, aged <2 years, were also underrepresented, preventing conclusions about this age group. Also, 94% of the cohort had a transvenous ICD system; therefore, the impact of other systems (epicardial and subcutaneous) on outcome could not be studied. Due to the excessive amount of missing data, we were unable to study ICD programming and its possible impact on the outcome, particularly inappropriate shocks. We did not study the impact of the different types of ICD manufacturers on outcomes such as battery longevity. The psychological impact of carrying an ICD could not be studied either. The study’s strength lies in its use of a national cohort, which limits the effects of treatment practices unique to single centres, and in the Swedish personal ID number system, which ensured no patients were lost to FU and allowed us to establish each patient’s current vital status with certainty. Furthermore, the data are based on thorough reviews of all patients’ charts, rather than reported registry data.

## Conclusions

The results of our study show that appropriate shocks were more than twice as common in the youngest patient group, whereas adverse events were not more frequent than in the older age group. These findings underline the importance of applying universally accepted criteria and guidelines for ICD implantation even in younger patients. A considerable number of appropriate as well as inappropriate shocks were associated with inadequate medication or non-compliance with medication. This means that many shocks and life-threatening events could be avoided with a more active treatment regimen and optimized pharmacological treatment.

## Supplementary Material

euaf317_Supplementary_Data

## Data Availability

That the data underlying this article should not be shared publicly was a condition of our ethics approval to protect the privacy of the participating patients. Thus, we are unable to share any of the individual patient data, even in anonymized form.

## References

[1] European Heart Rhythm Association; Heart Rhythm Society; Zipes DP, Camm AJ, Borggrefe M, Buxton AE et al ACC/AHA/ESC 2006 guidelines for management of patients with ventricular arrhythmias and the prevention of sudden cardiac death: a report of the American College of Cardiology/American Heart Association Task Force and the European Society of Cardiology Committee for Practice Guidelines (Writing Committee to Develop Guidelines for Management of Patients With Ventricular Arrhythmias and the Prevention of Sudden Cardiac Death). J Am Coll Cardiol 2006;48:e247–346. 16949478 10.1016/j.jacc.2006.07.010

[2] Writing Committee M, Silka MJ, Shah MJ, Silva JNA, Balaji S, Beach CM et al 2021 PACES expert consensus statement on the indications and management of cardiovascular implantable electronic devices in pediatric patients: executive summary. Ann Pediatr Cardiol 2022;15:323–46. 36589659 10.4103/0974-2069.361245 PMC9802608

[3] Silka MJ, Shah MJ, Silva JNA, Balaji S, Beach CM, Benjamin MN et al 2021 PACES expert consensus statement on the indications and management of cardiovascular implantable electronic devices in pediatric patients: executive summary. Cardiol Young 2021;31:1717–37. 34796795 10.1017/S1047951121003395

[4] Zeppenfeld K, Tfelt-Hansen J, de Riva M, Winkel BG, Behr ER, Blom NA et al 2022 ESC Guidelines for the management of patients with ventricular arrhythmias and the prevention of sudden cardiac death. Eur Heart J 2022;43:3997–4126. 36017572 10.1093/eurheartj/ehac262

[5] Konemann H, Dagres N, Merino JL, Sticherling C, Zeppenfeld K, Tfelt-Hansen J et al Spotlight on the 2022 ESC guideline management of ventricular arrhythmias and prevention of sudden cardiac death: 10 novel key aspects. Europace 2023;25:euad091. 10.1093/europace/euad091 PMC10228619 37102266

[6] Silvetti MS, Colonna D, Gabbarini F, Porcedda G, Rimini A, D'Onofrio A et al New guidelines of pediatric cardiac implantable electronic devices: what is changing in clinical practice? J Cardiovasc Dev Dis 2024;11:99. 38667717 10.3390/jcdd11040099 PMC11050217

[7] Paul T, Krause U, Sanatani S, Etheridge SP. Advancing the science of management of arrhythmic disease in children and adult congenital heart disease patients within the last 25 years. Europace 2023;25:euad155. 10.1093/europace/euad155 PMC10450816 37622573

[8] Norrish G, Protonotarios A, Stec M, Boleti O, Field E, Cervi E et al Performance of the PRIMaCY sudden death risk prediction model for childhood hypertrophic cardiomyopathy: implications for implantable cardioverter-defibrillator decision-making. Europace 2023;25:euad330. 10.1093/europace/euad330 PMC10666656 37995093

[9] Raatikainen MJ, Arnar DO, Zeppenfeld K, Merino JL, Levya F, Hindriks G et al Statistics on the use of cardiac electronic devices and electrophysiological procedures in the European Society of Cardiology countries: 2014 report from the European Heart Rhythm Association. Europace 2015;17:i1–i75. 25616426 10.1093/europace/euu300

[10] Nichols M, Townsend N, Scarborough P, Rayner M. Cardiovascular disease in Europe: epidemiological update. Eur Heart J 2013;34:3028–34. 24014390 10.1093/eurheartj/eht356

[11] DeWitt ES, Triedman JK, Cecchin F, Mah DY, Abrams DJ, Walsh EP et al Time dependence of risks and benefits in pediatric primary prevention implantable cardioverter-defibrillator therapy. Circ Arrhythm Electrophysiol 2014;7:1057–63. 25262116 10.1161/CIRCEP.114.001569

[12] Link MS, Hill SL, Cliff DL, Swygman CA, Foote CB, Homoud MK et al Comparison of frequency of complications of implantable cardioverter-defibrillators in children versus adults. Am J Cardiol 1999;83:263–6. A5-6. 10073833 10.1016/s0002-9149(98)00834-0

[13] Thuraiaiyah J, Philbert BT, Jensen AS, Xing LY, Joergensen TH, Lim CW et al Implantable cardioverter defibrillator therapy in paediatric patients for primary vs. secondary prevention. Europace 2024;26:euae245. 10.1093/europace/euae245 PMC11440178 39345160

[14] Song MK, Uhm JS, Baek JS, Yoon JK, Na JY, Yu HT et al Clinical outcomes of implantable cardioverter-defibrillator in pediatric patients—a Korean multicenter study. Circ J 2021;85:1356–64. 33980762 10.1253/circj.CJ-20-0468

[15] Lewandowski M, Syska P, Kowalik I. Children and young adults treated with transvenous and subcutaneous implantable cardioverter-defibrillators: a 22-year single-center experience and new perspectives. Kardiol Pol 2020;78:869–74. 32631024 10.33963/KP.15469

[16] Silvetti MS, Tamburri I, Porco L, Saputo FA, Di Mambro C, Righi D et al A decade of insertable cardiac monitors with remote monitoring in pediatric patients. Rev Cardiovasc Med 2022;23:27. 35092219 10.31083/j.rcm2301027

[17] Dechert BE, Bradley DJ, Serwer GA, Dick Ii M, Lapage MJ. Implantable cardioverter defibrillator outcomes in pediatric and congenital heart disease: time to system revision. Pacing Clin Electrophysiol 2016;39:703–8. 27119790 10.1111/pace.12878

[18] Silvetti MS, Drago F, Di Carlo D, Placidi S, Brancaccio G, Carotti A. Cardiac pacing in paediatric patients with congenital heart defects: transvenous or epicardial? Europace 2013;15:1280–6. 23439868 10.1093/europace/eut029

[19] Fortescue EB, Berul CI, Cecchin F, Walsh EP, Triedman JK, Alexander ME. Patient, procedural, and hardware factors associated with pacemaker lead failures in pediatrics and congenital heart disease. Heart Rhythm 2004;1:150–9. 15851146 10.1016/j.hrthm.2004.02.020

[20] Einbinder T, Machtei A, Birk E, Schamroth Pravda N, Frenkel G, Amir G et al Low risk of inappropriate shock among pediatric patients with an implantable cardioverter defibrillator: a single center experience. Pediatr Cardiol 2024;45:1776–83. 37668692 10.1007/s00246-023-03280-0

[21] von Alvensleben JC, Dechert B, Bradley DJ, Fish FA, Moore JP, Pilcher TA et al Subcutaneous implantable cardioverter-defibrillators in pediatrics and congenital heart disease: a pediatric and congenital electrophysiology society multicenter review. JACC Clin Electrophysiol 2020;6:1752–61. 33357571 10.1016/j.jacep.2020.07.010

[22] Taketomo CK, Hodding JH, Kraus DM (eds). A Comprehensive Resource for All Clinicians Treating Pediatric and Neonatal Patients. Hudson, Ohio: Lexicomp; 2011.

[23] Le Bos PA, Pontailler M, Maltret A, Kraiche D, Gaudin R, Barbanti C et al Epicardial vs. transvenous implantable cardioverter defibrillators in children. Europace 2023;25:961–8. 36735263 10.1093/europace/euad015 PMC10062323

[24] Von Bergen NH, Atkins DL, Dick M 2nd, Bradley DJ, Etheridge SP, Saarel EV et al Multicenter study of the effectiveness of implantable cardioverter defibrillators in children and young adults with heart disease. Pediatr Cardiol 2011;32:399–405. 21210096 10.1007/s00246-010-9866-7

[25] Heersche JH, Blom NA, van de Heuvel F, Blank C, Reimer AG, Clur SA et al Implantable cardioverter defibrillator therapy for prevention of sudden cardiac death in children in the Netherlands. Pacing Clin Electrophysiol 2010;33:179–85. 20025697 10.1111/j.1540-8159.2009.02603.x

[26] Berul CI, Van Hare GF, Kertesz NJ, Dubin AM, Cecchin F, Collins KK et al Results of a multicenter retrospective implantable cardioverter-defibrillator registry of pediatric and congenital heart disease patients. J Am Coll Cardiol 2008;51:1685–91. 18436121 10.1016/j.jacc.2008.01.033

[27] Silvetti MS, Pazzano V, Verticelli L, Battipaglia I, Saputo FA, Albanese S et al Subcutaneous implantable cardioverter-defibrillator: is it ready for use in children and young adults? A single-centre study. Europace 2018;20:1966–73. 29939256 10.1093/europace/euy139

[28] Aykan HH, Karagoz T, Gulgun M, Ertugrul I, Aypar E, Ozer S et al Midterm results of implantable cardioverter defibrillators in children and young adults from a single center in Turkey. Pacing Clin Electrophysiol 2016;39:1225–39. 27620455 10.1111/pace.12954

[29] Atallah J, Erickson CC, Cecchin F, Dubin AM, Law IH, Cohen MI et al Multi-institutional study of implantable defibrillator lead performance in children and young adults: results of the Pediatric Lead Extractability and Survival Evaluation (PLEASE) study. Circulation 2013;127:2393–402. 23694966 10.1161/CIRCULATIONAHA.112.001120

[30] Silvetti MS, Bruyndonckx L, Maltret A, Gebauer R, Kwiatkowska J, Kornyei L et al The SIDECAR project: S-IcD registry in European paediatriC and young Adult patients with congenital heaRt defects. Europace 2023;25:460–8. 36107451 10.1093/europace/euac162 PMC9935000

[31] Migliore F, Biffi M, Viani S, Pittorru R, Francia P, Pieragnoli P et al Modern subcutaneous implantable defibrillator therapy in patients with cardiomyopathies and channelopathies: data from a large multicentre registry. Europace 2023;25:euad239. 37536671 10.1093/europace/euad239 PMC10438213

[32] Taddeo D, Egedy M, Frappier JY. Adherence to treatment in adolescents. Paediatr Child Health 2008;13:19–24. 19119348 10.1093/pch/13.1.19 PMC2528818

