# Supplementary material for: Outcomes for patients implanted with a cardioverter-defibrillator at <19 years of age: a Swedish national study
Source: Europace. 2025 Dec 11;28(1):euaf317. doi: 10.1093/europace/euaf317 (PMC12849815; doi:10.1093/europace/euaf317)
Supplement: euaf317_Supplementary_Data [file euaf317_supplementary_data.zip › Supplementary tables and figure legends.docx]

Supplementary table 1. Characteristics of 16 patients with lower weight at implantation

| Patient | Diagnosis | Age  (y) | Follow-up (y) | Indication  (P/S) | Appropriate shocks | Inappropriate shocks | Complications  (Y/N) | First ICD-system  (E/T) |
| --- | --- | --- | --- | --- | --- | --- | --- | --- |
| 1 | CPVT | 1.1 | 11.2 | S | Y | N | Y | E |
| 2 | CPVT | 3.1 | 9.4 | S | Y | N | Y | E |
| 3 | DCM | 9 | 4.2 | S | Y | N | N | T |
| 4 | DCM | 10.4 | 7.1 | S | Y | N | Y | T |
| 5 | HCM | 6.2 | 4.9 | P | N | N | Y | E |
| 6 | HCM | 7.5 | 6.1 | P | Y | N | N | T |
| 7 | HCM | 7.7 | 10.7 | P | N | Y | N | T |
| 8 | HCM | 7.5 | 8.7 | P | N | N | N | T |
| 9 | HCM | 7.7 | 10.7 | P | N | Y | Y | T |
| 10 | HCM | 10.7 | 5.8 | P | N | N | N | T |
| 11 | HCM | 11.4 | 4.4 | S | Y | N | N | T |
| 12 | LQTS | 5.7 | 9.8 | S | Y | N | N | E |
| 13 | LQTS | 6.2 | 1.9 | S | N | N | N | T |
| 14 | LQTS | 8.6 | 6.9 | S | Y | N | N | T |
| 15 | LQTS | 10.6 | 10.3 | S | Y | N | N | T |
| 16 | LQTS | 10.2 | 15.5 | S | Y | N | Y | T |

Age and follow-up in years (y). Y; yes, N; no. CPVT; catecholaminergic polymorphic ventricular tachycardia, DCM; dilated cardiomyopathy, HCM; hypertrophic cardiomyopathy, LQTS; long QT syndrome, E; epicardial ICD, T; transvenous ICD

Supplementary table 2. Cox regression. ICD system survival according to indication, disease category, weight group and cohort.

|  | Coefficient | HR | SE(coefficient) | z-value | p-value |
| --- | --- | --- | --- | --- | --- |
| **Indication**- secondary vs. primary | 0.443 | 1.557 | 0.296 | 1.497 | 0.134 |
| **Disease category**- CMP vs. PED | 0.088 | 1.092 | 0.300 | 0.295 | 0.768 |
| **Disease category**- other vs. PED | 0.064 | 1.066 | 0.503 | 0.127 | 0.899 |
| **Weight group**- lower-weight vs. higher-weight | 0.343 | 1.410 | 0.356 | 0.964 | 0.335 |
| **Cohort**- late vs. early era | -0.771 | 0.462 | 0.307 | -2.512 | **0.012** |

CMP, cardiomyopathy; PED, primary electrical disease; HR, hazard ratio.

Supplementary table 3. Cox regression. Survival without appropriate shocks according to indication, disease category, weight group and cohort.

|  | Coefficient | HR | SE(coefficient) | z-value | p-value |
| --- | --- | --- | --- | --- | --- |
| **Indication**- secondary vs. primary | 1.319 | 3.740 | 0.411 | 3.210 | **0.001** |
| **Disease category**- CMP vs. PED | 0.353 | 1.424 | 0.360 | 0.980 | 0.327 |
| **Disease category**- other vs. PED | -1.512 | 0.220 | 1.035 | -1.461 | 0.144 |
| **Weight group**- lower-weight vs. higher-weight | 0.996 | 2.706 | 0.374 | 2.661 | **0.008** |
| **Cohort**- late vs. early era | -0.118 | 0.888 | 0.341 | -0.348 | 0.728 |

CMP, cardiomyopathy; PED, primary electrical disease; HR, hazard ratio.

Supplementary table 4. Cox regression. Survival without inappropriate shocks according to indication, disease category, weight group and cohort.

|  | Coefficient | HR | SE(coefficient) | z-value | p-value |
| --- | --- | --- | --- | --- | --- |
| **Indication**- secondary vs. primary | 0.339 | 1.404 | 0.422 | 0.803 | 0.422 |
| **Disease category**- CMP vs. PED | 0.757 | 2.131 | 0.461 | 1.640 | 0.101 |
| **Disease category**- other vs. PED | 0.423 | 1.527 | 0.688 | 0.615 | 0.538 |
| **Weight group**- lower-weight vs. higher-weight | -0.701 | 0.496 | 0.745 | -0.941 | 0.347 |
| **Cohort**- late vs. early era | -0.811 | 0.444 | 0.461 | -1.759 | 0.079 |

CMP, cardiomyopathy; PED, primary electrical disease; HR, hazard ratio.

Supplementary table 5. Cox regression. Survival without complications according to indication, disease category, weight group and cohort.

|  | Coefficient | HR | SE(coefficient) | z-value | p-value |
| --- | --- | --- | --- | --- | --- |
| **Indication**- secondary vs. primary | -0.300 | 0.741 | 0.380 | -0.788 | 0.431 |
| **Disease category**- CMP vs. PED | -0.175 | 0.839 | 0.382 | -0.459 | 0.646 |
| **Disease category**- other vs. PED | -18.303 | 0.000 | 4078.484 | +0.004 | 0.996 |
| **Weight group**- lower-weight vs. higher-weight | 0.121 | 1.129 | 0.456 | 0.266 | 0.790 |
| **Cohort**- late vs. early era | -0.090 | 0.914 | 0.405 | -0.221 | 0.825 |

CMP, cardiomyopathy; PED, primary electrical disease; HR, hazard ratio.

Supplementary table 6. Characteristics of patients who died.

| Patient | Diagnosis | Age at death  (y) | Follow-up  (y) | Sex  (M/F) | Appropriate  shocks | Inappropriate  shocks | Complications  (Y/N) | C O D |
| --- | --- | --- | --- | --- | --- | --- | --- | --- |
| 1 | ARVC | 17 | 0.3 | M | 1 | 0 | N | Arrhythmia |
| 2 | CHD | 36 | 17.9 | M | 1 | 0 | N | Heart failure |
| 3 | CPVT | 21 | 4.1 | M | 0 | 0 | Y | U - ICD explanted |
| 4 | DCM | 11 | 1 | F | 1 | 0 | Y | Arrhythmia |
| 5 | DCM | 13 | 4.2 | M | 3 | 0 | N | Arrhythmia |
| 6 | HCM | 16 | 5.8 | F | 0 | 0 | N | U - SCD abroad |
| 7 | HCM | 19 | 4.4 | M | 0 | 2 | N | M O F |
| 8 | HCM | 21 | 5.9 | M | 6 | 1 | Y | Arrhythmia, M O F |

Values are age, follow-up in years (y) or number (n) of shocks. Y; yes, N; no, U; unknown, C O D; cause of death, ICD; implantable cardiac defibrillator, M O F; multi organ failure, ARVC; arrhythmogenic right ventricular cardiomyopathy, CPVT; catecholaminergic polymorphic ventricular tachycardia, DCM; dilated cardiomyopathy, HCM; hypertrophic cardiomyopathy.

**Supplementary Figure legend 1.** The incidence rate ratio (**IRR**) of ICD implantations comparing adults to young individuals is shown for each time period. The incidence (**Iadult**) in the adult population (age ≥19 years at implantation) is compared to the incidence **(Iyoung)** in the young population (age <19 years at implantation) and calculated as: IRR= Iadult/Iyoung. The value for each time period is expressed as the average of the included years.

**Supplementary Figure legend 2**. Antiarrhythmic medication and compliance in connection with any delivered shock divided in (A) appropriate shocks and (B) inappropriate shocks. The events are grouped according to whether there was adequate/inadequate medication, or no medication, and whether there was compliance or non-compliance with medication, respectively.

**Supplementary Figure legend 3**. ICD system survival according to (A) cohort era (ICD implanted <2010 vs. ≥2010) and (B) patient’s weight at implantation (lower-weight vs. higher-weight).

**Supplementary Figure legend 4**. Probability of freedom from inappropriate shocks. Patients are compared with respect to (A) underlying disease category (primary electrical disease vs. cardiomyopathies vs. others), and (B) cohort era at the time of ICD implantation.
