# Supplementary figures and images for: Outcomes for patients implanted with a cardioverter-defibrillator at <19 years of age: a Swedish national study
Source: Europace. 2025 Dec 11;28(1):euaf317. doi: 10.1093/europace/euaf317 (PMC12849815; doi:10.1093/europace/euaf317)

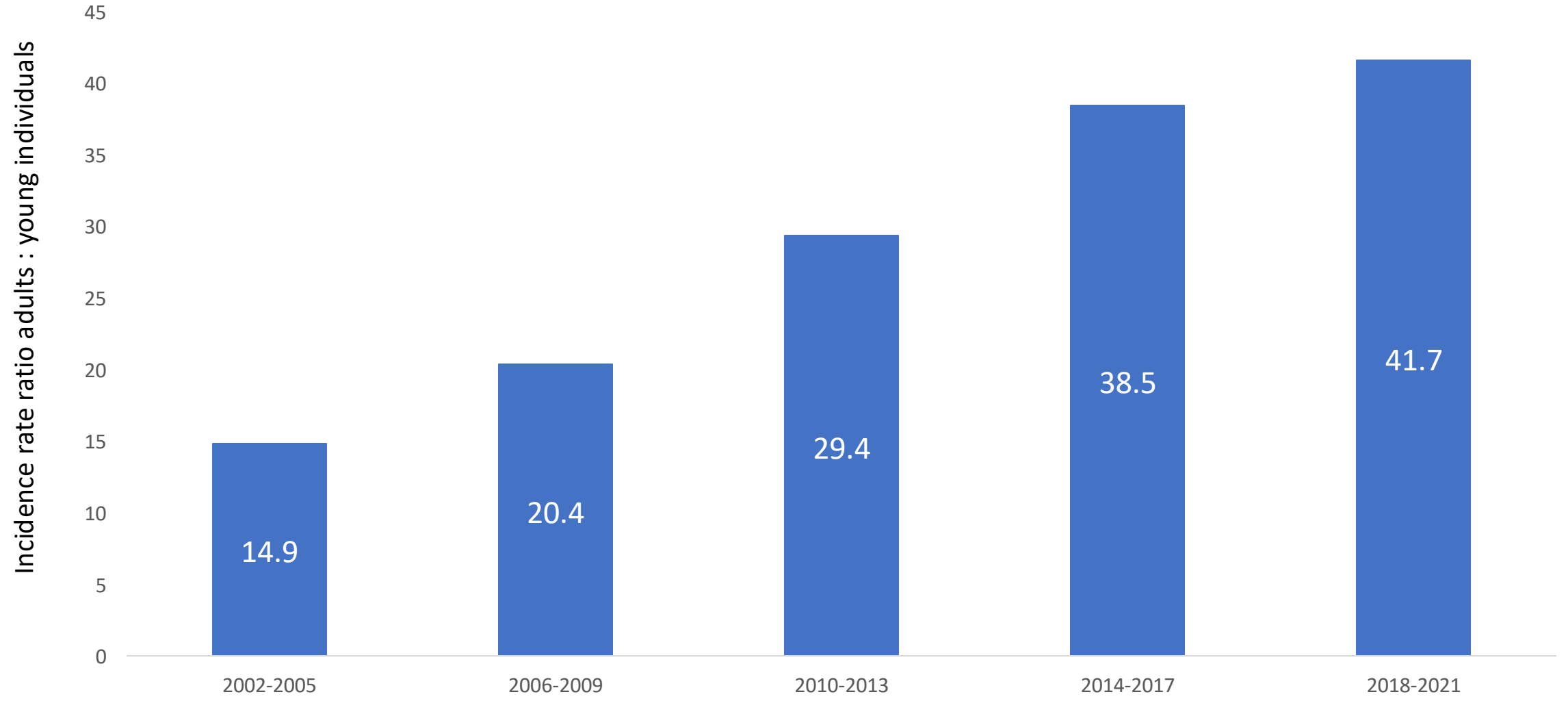

Supplement: euaf317_Supplementary_Data [file euaf317_supplementary_data.zip › Supplementary figure 1. Incidence rate ratio of ICD.pdf]

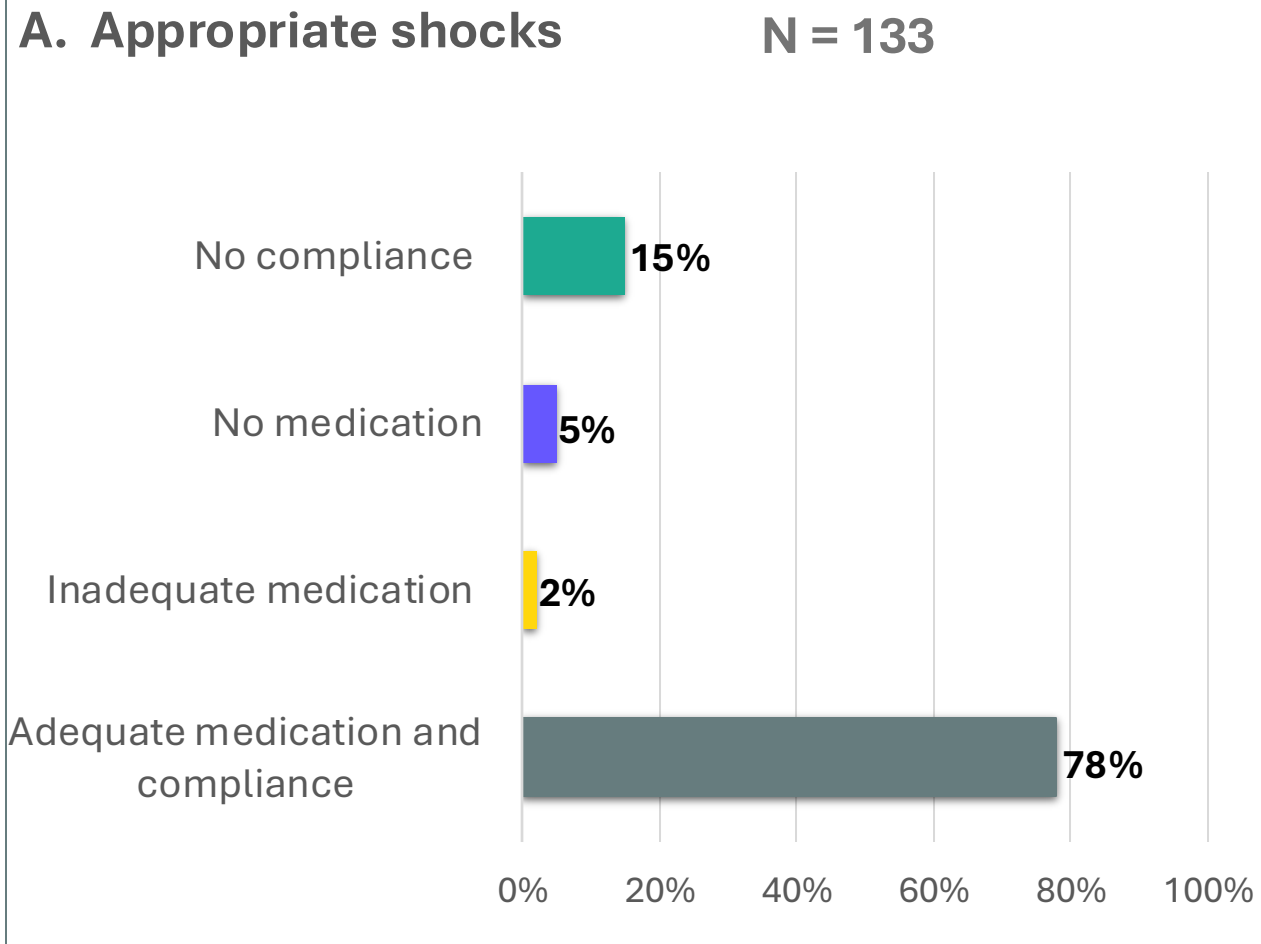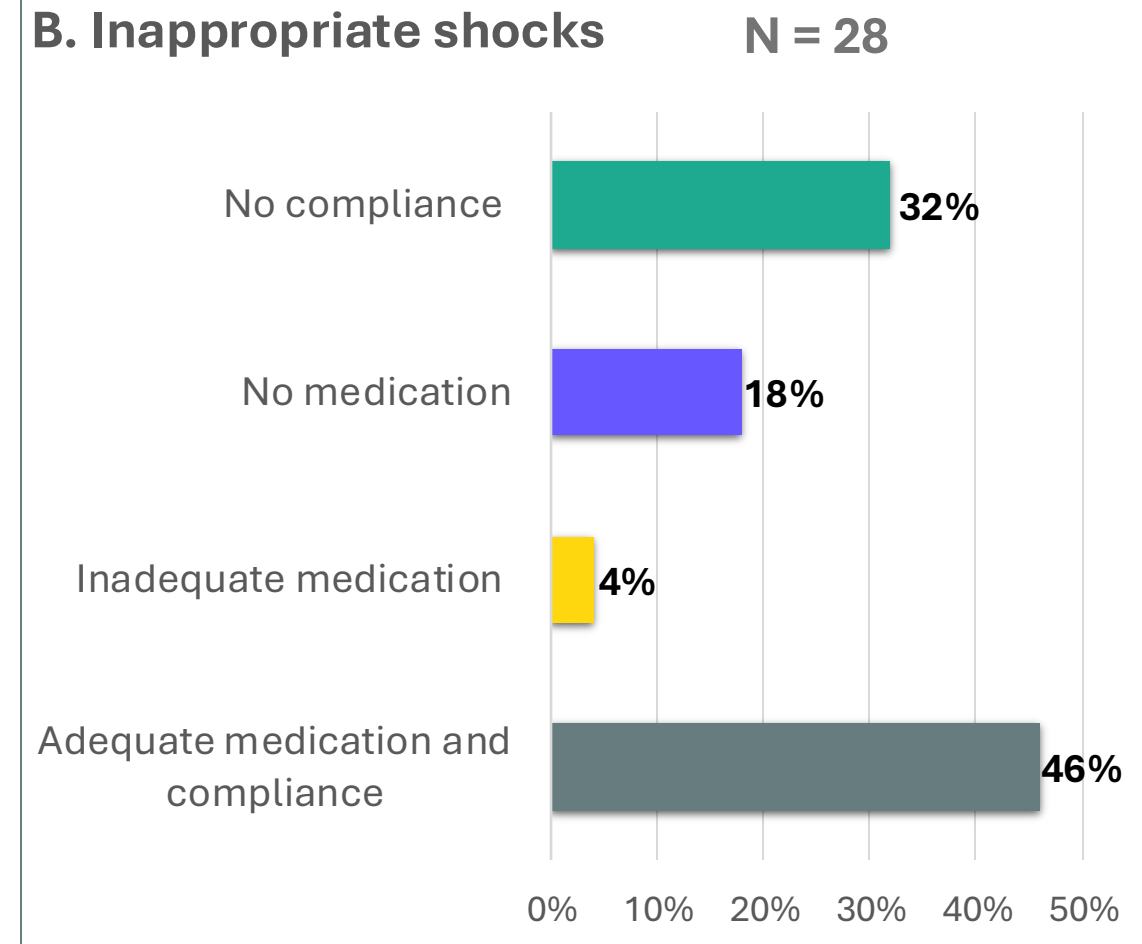

Supplement: euaf317_Supplementary_Data [file euaf317_supplementary_data.zip › Supplementary Figure 2. Antiarrhythmic medication and shocks.pdf]

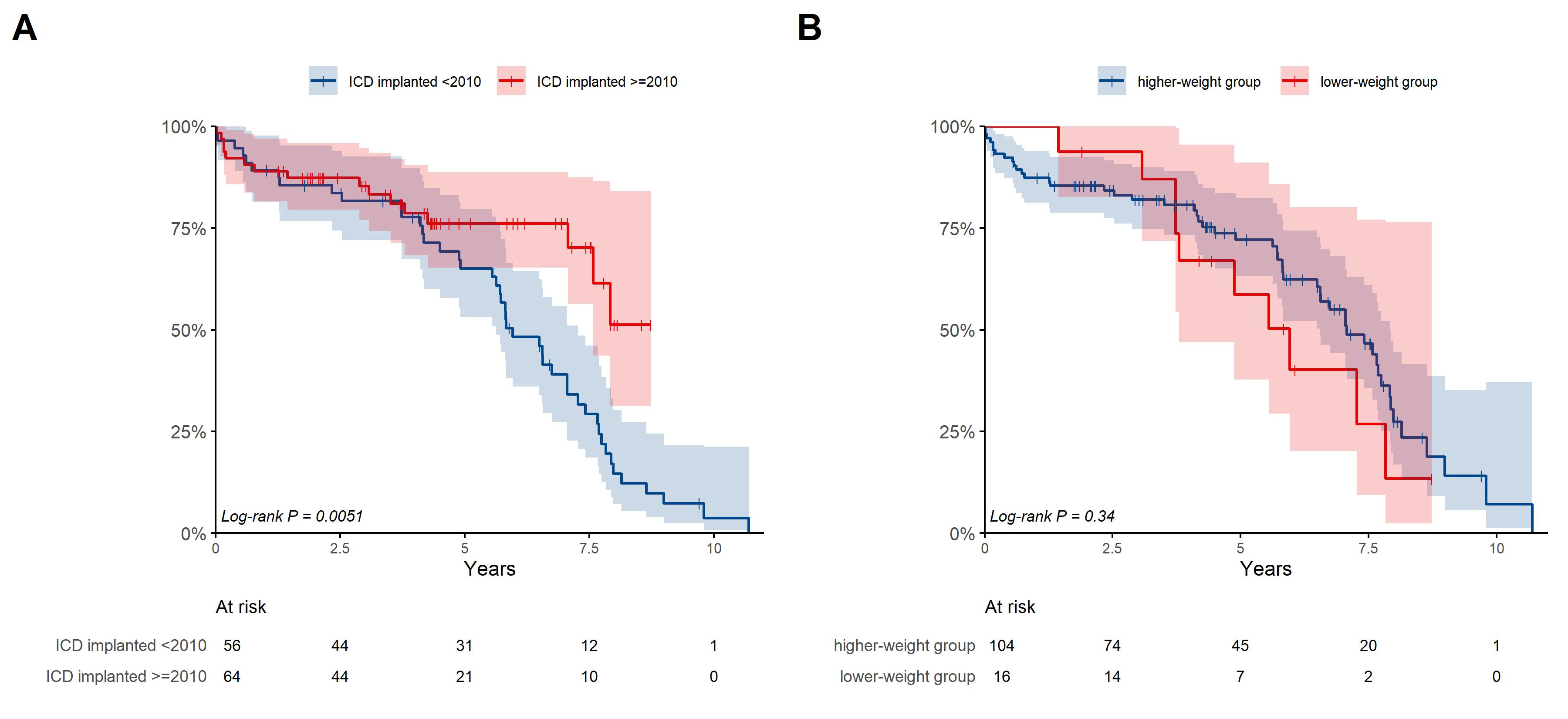

Supplement: euaf317_Supplementary_Data [file euaf317_supplementary_data.zip › Supplementary Figure 3. ICD system survival .jpeg]

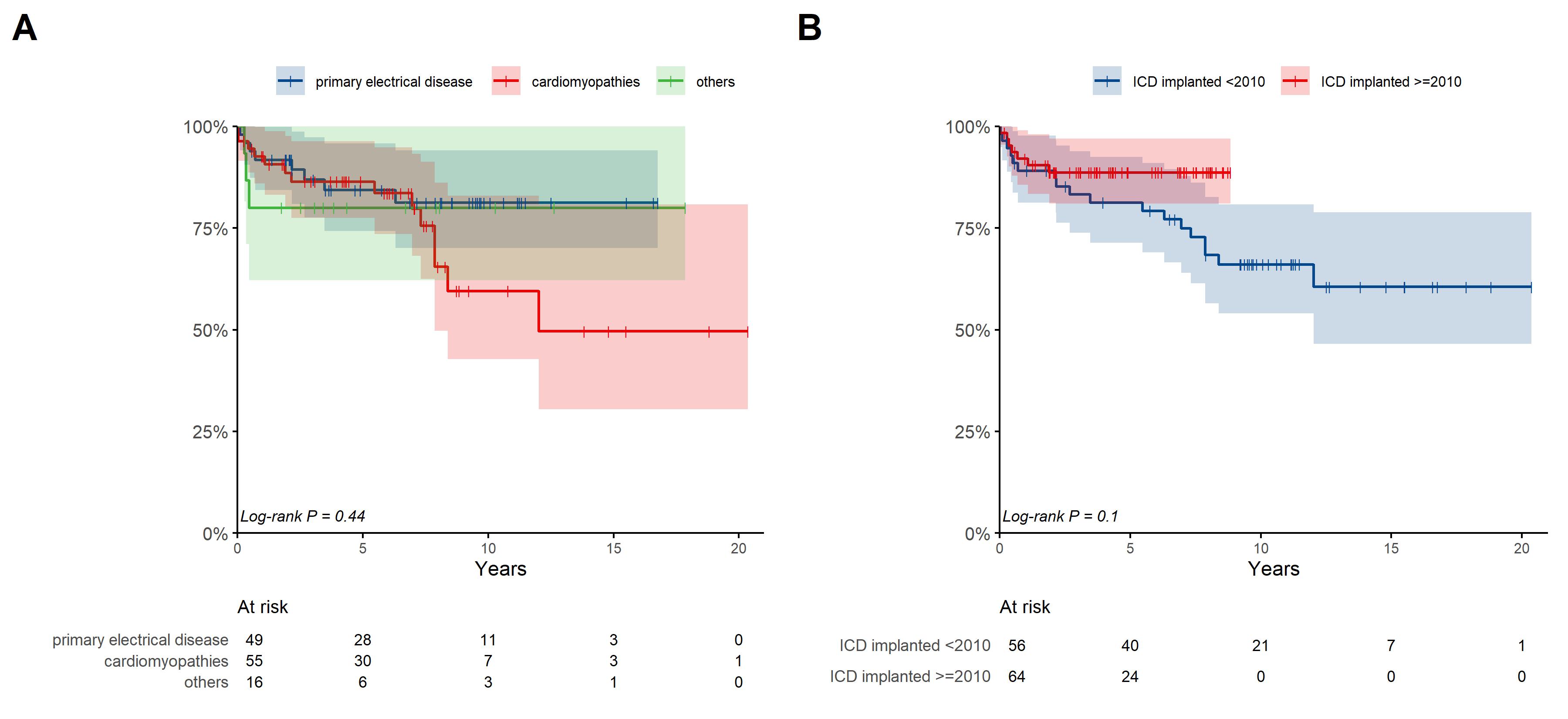

Supplement: euaf317_Supplementary_Data [file euaf317_supplementary_data.zip › Supplementary Figure 4. Freedom from inappropriate shocks.jpeg]
